# Identification of potential models for predicting progestin insensitivity in patients with endometrial atypical hyperplasia and endometrioid endometrial cancer based on ATAC-Seq and RNA-Seq integrated analysis

**DOI:** 10.3389/fgene.2022.952083

**Published:** 2022-08-26

**Authors:** Jia-Li Hu, Gulinazi Yierfulati, Lu-Lu Wang, Bing-Yi Yang, Qiao-Ying Lv, Xiao-Jun Chen

**Affiliations:** ^1^ Department of Gynecology, Obstetrics and Gynecology Hospital of Fudan University, Shanghai, China; ^2^ Shanghai Key Laboratory of Female Reproductive Endocrine Related Diseases, Shanghai, China

**Keywords:** predictive models, progestin insensitivity, endometrial atypical hyperplasia, endometrioid endometrial cancer, ATAC-seq, RNA-seq, fertility-preserving treatment

## Abstract

**Objective:** The aim of this study was to establish predictive models based on the molecular profiles of endometrial lesions, which might help identify progestin-insensitive endometrial atypical hyperplasia (EAH) or endometrioid endometrial cancer (EEC) patients before progestin-based fertility-preserving treatment initiation.

**Methods:** Endometrial lesions from progestin-sensitive (PS, *n* = 7) and progestin-insensitive (PIS, *n* = 7) patients were prospectively collected before progestin treatment and then analyzed by ATAC-Seq and RNA-Seq. Potential chromatin accessibility and expression profiles were compared between the PS and PIS groups. Candidate genes were identified by bioinformatics analyses and literature review. Then expanded samples (*n* = 35) were used for validating bioinformatics data and conducting model establishment.

**Results:** ATAC-Seq and RNA-Seq data were separately analyzed and then integrated for the subsequent research. A total of 230 overlapping differentially expressed genes were acquired from ATAC-Seq and RNA-Seq integrated analysis. Further, based on GO analysis, REACTOME pathways, transcription factor prediction, motif enrichment, Cytoscape analysis and literature review, 25 candidate genes potentially associated with progestin insensitivity were identified. Finally, expanded samples were used for data verification, and based on these data, three predictive models comprising 9 genes (*FOXO1*, *IRS2*, *PDGFC*, *DIO2*, *SOX9*, *BCL11A*, *APOE*, *FYN*, and *KLF4*) were established with an overall predictive accuracy above 90%.

**Conclusion:** This study provided potential predictive models that might help identify progestin-insensitive EAH and EEC patients before fertility-preserving treatment.

## Introduction

Endometrioid endometrial cancer (EEC) is one of the most common gynecological malignancies, with an increasing trend in new cancer cases and deaths each year (Siegel et al., 2022). Notably, EEC and its precancerous lesions, endometrial atypical hyperplasia (EAH), present a younger trend, and approximately half of young EEC and EAH patients are nulliparous when diagnosed (Trojano et al., 2019). Therefore, fertility-sparing treatment for these patients has attracted increasing attention in clinical research. Currently, high-dose progestin therapy is the main conservative strategy and achieves an approximately 70–80% complete response (CR) rate, and the median duration from treatment to CR is as long as six to 7 months (Gallos et al., 2012; Gunderson et al., 2012; Yang et al., 2019; Westin et al., 2021). However, there are still approximately 20–30% of cases are not sensitive to progestin and having to switch to second-line treatment or even receive definitive surgery. Identifying progestin-insensitive (PIS) cases accurately before progestin treatment initiation might aid clinicians in providing more efficient treatment for these patients and thus improve the overall outcome of fertility-preserving treatment.

There is still a lack of objective indicators predicting progestin sensitivity in EAH or EEC patients. Studies have shown that positive progesterone receptor (PR) expression in EAH and EEC tissues was associated with shorter CR time of fertility-sparing therapy (Yamazawa et al., 2007; Raffone et al., 2019; Wang et al., 2021). While the abnormal expression of other molecular markers, such as elevated dual-specificity phosphatase 6 or downregulated nuclear factor NF-E2-related factor or survivin, might be associated with progestin insensitivity (Zhang et al., 2015; Fan et al., 2017). However, there is less high-quality evidence of molecular markers that can be used to predict progestin response in EAH and EEC cases. Therefore, further studies are still needed to explore promising models for predicting progestin response in EAH and EEC cases.

To explore potential predictive models for predicting progestin insensitivity in EAH or EEC patients before receiving progestin-based fertility-preserving treatment, this study was designed based on assay for transposase-accessible chromatin sequencing (ATAC-Seq) and RNA sequencing (RNA-Seq) of EAH and EEC tissues. Based on ATAC-Seq and RNA-Seq integrated bioinformatics analyses and literature review, candidate genes were identified and further verified in another 35 cases for predictive model construction. Our study provided potential models for predicting progestin insensitivity in patients with EAH and EEC.

## Materials and methods

### Ethics statement

This is a retrospective study using samples prospectively collected from December 2017 to November 2020, in the Obstetrics and Gynecology Hospital of Fudan University, Shanghai, China (hereafter referred to as ‘Ob&Gyn Hospital’). This study was approved by the Ethics Committees of Ob&Gyn Hospital (Approval NO. 2021-130). Patients were fully informed of the use of their medical data and pathological samples for scientific research, and signed informed consent forms.

### Patient selection and tissue collection

Young patients diagnosed with EAH or well-differentiated EEC receiving progestin-based fertility-sparing treatment were prospectively registered. All patients were pathologically diagnosed with EAH or EEC for the first time by endometrial biopsy with or without hysteroscopy. Inclusion and exclusion criteria as well as treatment regimen and evaluation procedure were as previously reported (Yang et al., 2020). Briefly, patients received progestin-based treatment, hysteroscopic evaluation and endometrial biopsy every 3 months on average. Pathological diagnosis was confirmed by at least two experienced gynecological pathologists independently according to the World Health Organization (WHO) pathological classification (2020). If their opinions differed, a seminar was held in the pathological department for the final diagnosis.

‘PIS’ was defined as disease progression at any time during treatment, stable disease after 7 months of treatment, or did not achieve CR after 10 months of treatment (Zhou and Xu, 2021). Other patients who achieved CR within 10 months of treatment were regarded as ‘PS'.

Endometrial lesions before progestin treatment initiation were prospectively collected through biopsy under hysteroscopy and stored at -80°C equipped with or without RNA preservation solution. Samples from 7 PIS patients and 7 PS patients were firstly collected for ATAC-Seq and RNA-Seq analyses from December 2017 to November 2019 (regarded as the ‘Analysis Group’). Because the number of EAH or EEC patients receiving fertility preserving treatment is relatively low, we tried to collect as many patients as possible for validation to minimize possible bias caused by low case number. As a result, a total of 35 cases met the inclusion and exclusion criteria of this study were recruited from November 2019 to November 2020. These patients were regarded as ‘Construction Group’ for validation and model construction. They were further classified as PS-C (achieved CR within 5 months of treatment, *n* = 13), sub-PS-C (achieved CR within 5–9 months of treatment, *n* = 15) and PIS-C (*n* = 7). The basic characteristics of the enrolled patients were shown in Table 1.

**TABLE 1:** General characteristics of the study population.

Variables	Analysis group				Construction group				
	Total	PS	PIS	∗*p* value	Total	PS-C	sub-PS-C	PIS-C	^+^ *p* value
Patients (n)	14	7	7	—	35	13	15	7	—
Diagnosis									1.000
EAH	7 (50)	4 (57.1)	4 (57.1)	1.000	25 (69.44)	10 (76.92)	10 (66.67)	5 (62.5)	—
EEC	7 (50)	3 (42.9)	3 (42.9)		11 (30.56)	3 (23.08)	5 (33.33)	3 (37.5)	—
Age at diagnosis (year)	31 (26–36)	34 (28–36)	30 (26–34)	0.097	32.5 (21–42)	34 (21–39)	30 (23–36)	34 (24–42)	0.2895
BMI (kg/m2)	28.26 (20.70–37.65)	28.13 (23.44–36.13)	28.40 (20.70–37.65)	0.710	28.09 (18.87–45.17)	26.15 (18.87–37.74)	28.04 (19.57–45.17)	29.94 (20.28–35.26)	0.880
HOMA-IR	4.15 (1.40–6.37)	4.41 (1.47–6.37)	3.53 (1.40–5.58)	0.535	3.16 (0.84–22.80)	4.12 (1.18–10.13)	3.23 (0.84.22.80)	2.35 (1.56–7.64)	0.647
MS^§^	8 (57.1)	4 (57.1)	4 (57.1)	1.000	15 (41.7)	5 (38.5)	6 (40.0)	4 (50.0)	0.830
Hypertension	3 (21.4)	2 (28.6)	1 (14.3)	1.000	3 (8.3)	1 (7.7)	2 (13.3)	0 (0.0)	0.782
Diabetes mellitus	0 (0.0)	0 (0.0)	0 (0.0)	—	4 (11.1)	1 (7.7)	2 (13.3)	1 (12.5)	1.000
Nulliparous	11 (78.6)	5 (71.4)	6 (85.7)	1.000	29 (80.6)	9 (69.2)	13 (86.7)	7 (87.5)	0.553
Progestin therapy									
MA	6	2 (28.6)	4 (57.1)		12 (33.3)	2 (15.4)	8 (53.3)	2 (25.0)	
MA + Metformin	4	2 (28.6)	2 (28.6)		12 (33.3)	4 (30.8)	6 (40.0)	2 (25.0)	
LNG-IUD	1	1 (14.3)	0 (0)		4 (11.1)	3 (23.1)	0 (0.0)	1 (12.5)	
MA + LNG-IUD	3	2 (28.6)	1 (14.3)		4 (11.11)	2 (15.4)	0 (0.0)	2 (25.0)	
MA + Rosuvastatin	-	-	-		4 (11.1)	2 (15.4)	1 (6.7)	1 (12.5)	
CR time (months)^††^	7.8 (3.7–29.5)	7.0 (3.7–7.9)	12.0 (6.0–29.5)	0.011	6.33 (3.07–13.23)	3.9 (3.07–4.90)	6.87 (5.87–8.1)	11.17 (10.53–13.23)^‡‡^	<0.0001

†† Total treatment duration from initiation of conservative treatment to CR.

^‡‡^Note: CR time of one patient in PIS-C group was not included, because this patient did not achieve CR and underwent hysterectomy eventually.

^§^Diagnosis of MS meets at least three of the following criteria: 1) BP ≥ 130/85 mmHg or hypertension; 2) Waist circumference ≥80 cm; 3) Total cholesterol ≥1.7 mmol/L; 4) High density lipoprotein <1.04 mmol/L; 5) Fasting plasma glucose ≥5.6 mmol/L or type II diabetes mellitus.

∗*p* value: comparison between PS group and PIS group in Analysis Group.

^+^ *p* value: comparison between PS-C group, sub-PS-C group and PIS-C group in Construction Group.

Values are presented as median (range) or number (%).

PS, progestin-sensitive; PIS, progestin-insensitive; PS-C, progestin-sensitive in Construction Group; sub-PS-C, progestin-sub-sensitive in Construction Group; PIS-C, progestin-insensitive in Construction Group; EAH, endometrial atypical hyperplasia; EEC, endometrioid endometrial cancer; BMI, body mass index; HOMA-IR, homeostasis model assessment-insulin resistance; MS, metabolic syndrome; MA, megestrol acetate; LNG-IUD, levonorgestrel intrauterine device; CR, complete response.

### Library construction and ATAC-Seq analysis

ATAC-Seq was performed to analyze transposase accessible chromatin as previously described (Buenrostro et al., 2015). An improved ATAC-Seq protocol that reduces background and enables interrogation of frozen tissues was used for nuclei collection (Corces et al., 2017). Libraries were pooled at equimolar ratios with barcodes and sequenced on the BGISEQ-500 platform (BGI, Shenzhen, China).

Raw sequence reads were initially processed for quality control by FastQC. Before statistical analysis, ATAC-Seq read counts of different samples were normalized according to the methods described previously (Zhang and Parmigiani, 2020). In ATAC-Seq analysis, opening or closing peaks were chosen with |log_2_ fold change|>0.5849 and non-adjusted *p* < 0.05 (PIS vs. PS). The proportion of all reads in each sample was matched to the elements in the human genome according to functional and positional information, including 3′ UTR, 5′UTR, distal intergenic, downstream, exon, intron, and promoter. Scatter plot showed the accessibility at each peak. Hierarchical cluster analysis was performed to assess chromatin accessibility with differential gene peaks.

### Library construction and RNA-Seq analysis

RNA-Seq was performed to assess the expression of genes in tissue samples as described previously (Wang et al., 2018a). Libraries were generated on the BGIseq500 platform (BGI-Shenzhen, China). Fragments per kilobase per million reads (FPKM) was used to quantitatively estimate gene expression values (Trapnell et al., 2010). DESeq2 was used to analyze the raw count (Wang et al., 2010). Before statistical analysis, RNA-Seq read counts of different samples were normalized according to a previously reported method (Zhang and Parmigiani, 2020). Differential expression analysis was performed using the R DESeq2 package (v1.4.5) (Love et al., 2014). Genes with |log_2_ fold change|>0.5849 and non-adjusted *p* < 0.05 (PIS vs. PS) were defined as differentially expressed genes (DEGs) between the PIS and PS patients. A heatmap was drawn to cluster the DEGs. The DEGs were further analyzed by Gene Ontology (GO) and REACTOME pathways to determine the potential functions and pathways enriched by these DEGs using R packages. GO analysis included biological process (BP), molecular function (MF), and cellular components (CC).

### Integration analysis of ATAC-Seq and RNA-Seq

ATAC-Seq and RNA-Seq profiles were analyzed after integration to accurately determine the potential center genes that can distinguish PIS from PS patients. The overlapping DEGs were defined as 1) the upregulated DEGs in RNA-Seq with an enhanced chromatin open region signal in ATAC-Seq and 2) the downregulated DEGs in RNA-Seq with an attenuated chromatin open region signal in ATAC-Seq (PIS vs. PS). A Venn diagram was generated to present the overlapping upregulated and downregulated DEGs. Scatter plots were used to evaluate the relationship between the transposase accessible chromatin and gene expression derived from ATAC-Seq and RNA-Seq data, respectively.

The candidate genes for predictive model construction were screened out based on ATAC-Seq and RNA-Seq integrated bioinformatics analyses and literature review, but not only based on the level of change between the two conditions. The bioinformatics analyses in this part included REACTOME pathways, Transcription factor (TF) prediction, Motif enrichment, and Functional protein-associated networks. 1) Based on overlapping DEGs by ATAC-Seq and RNA-Seq integrated analysis, top ten REACTOME pathways were enriched, and DEGs in the pathways potentially regulating progestin insensitivity were first screened out. 2) Potential TFs that regulate the expression of the overlapping DEGs were enriched by HOMER Software, and DEGs-encoding TFs with *p* value less than 0.05 were screened out. 3) Motif enrichment was performed to identify important TFs by using homer peak analysis software. The generated homer known TFs with *p* value less than 0.05 and more than 20% of target sequences with motifs enriched in chromatin regions were listed in Table 2, and their encoding genes among the overlapping DEGs were identified. 4) The interactions between proteins encoded by overlapping DEGs were analyzed using STRING (https://string-db.org/) and Cytoscape software (version 3.6.1). Central proteins were determined with both >4 connected lines and >0.4 combined score, and their encoding DEGs were identified. Furthermore, all the candidate genes screened out based on aforementioned bioinformatics analyses above, were comprehensively evaluated by literature review according to whether these candidate genes were involved in tumor initiation, progression and treatment resistance.

**TABLE 2:** TFs binding homer known motifs enriched in chromatin region in response to progestin in PIS group compared to PS group from Analysis Group.

TFs	Binding motif	% Of target sequences with motif	*p* Value
NANOG	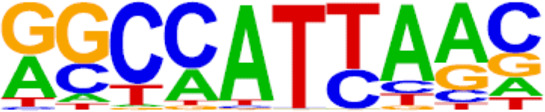		
	Nanog (Homeobox)/mES-Nanog-ChIP-Seq (GSE11724)/Homer	44.87%	1.00E−02
TGIF2	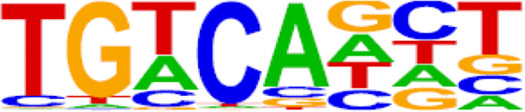		
	Tgif2 (Homeobox)/mES-Tgif2-ChIP-Seq (GSE55404)/Homer	39.74%	1.00E−02
NF1	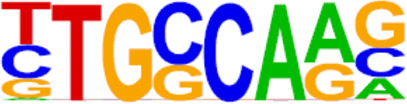		
	NF1-halfsite (CTF)/LNCaP-NF1-ChIP-Seq (Unpublished)/Homer	29.49%	1.00E−05
HOXA9	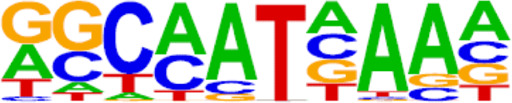		
	Hoxa9 (Homeobox)/ChickenMSG-Hoxa9.Flag-ChIP-Seq (GSE86088)/Homer	29.17%	1.00E−02
FOXO1	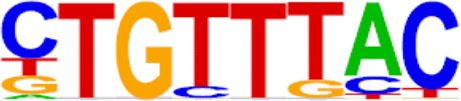		
	Foxo1 (Forkhead)/RAW-Foxo1-ChIP-Seq (Fan_et_al.)/Homer	28.53%	1.00E−06
SP2	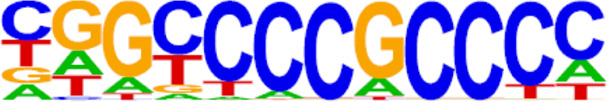		
	Sp2 (Zf)/HEK293-Sp2.eGFP-ChIP-Seq (Encode)/Homer	28.53%	1.00E−03
SOX10	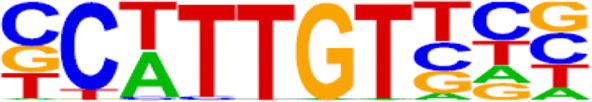		
	Sox10 (HMG)/SciaticNerve-Sox3-ChIP-Seq (GSE35132)/Homer	26.60%	1.00E−08
SOX3	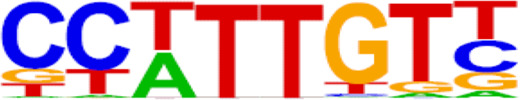		
	Sox3 (HMG)/NPC-Sox3-ChIP-Seq (GSE33059)/Homer	25.64%	1.00E−06
TWIST2	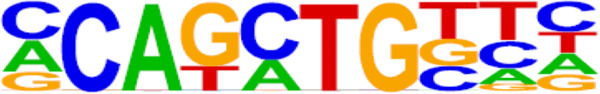		
	Twist2 (bHLH)/Myoblast-Twist2.Ty1-ChIP-Seq (GSE127998)/Homer	25.64%	1.00E−03
SOX6	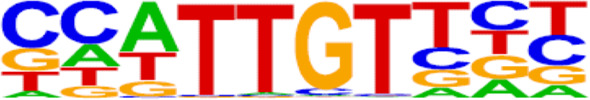		
	Sox6 (HMG)/Myotubes-Sox6-ChIP-Seq (GSE32627)/Homer	25.00%	1.00E−07
SOX21	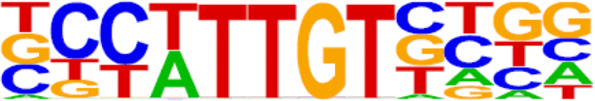		
	Sox21 (HMG)/ESC-SOX21-ChIP-Seq (GSE110505)/Homer	24.68%	1.00E−04
KLF5	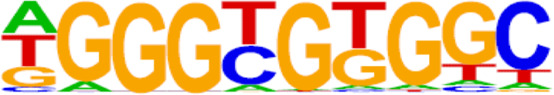		
	KLF5 (Zf)/LoVo-KLF5-ChIP-Seq (GSE49402)/Homer	23.72%	1.00E−02
MAZ	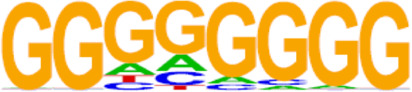		
	Maz (Zf)/HepG2-Maz-ChIP-Seq (GSE31477)/Homer	23.08%	1.00E−02
TCF4	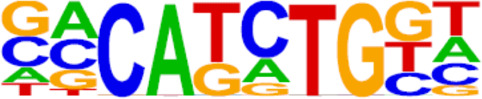		
	TCF4 (bHLH)/SHSY5Y-TCF4-ChIP-Seq (GSE96915)/Homer	22.76%	1.00E−03
AP-1	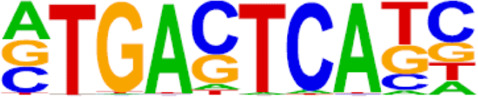		
	AP-1 (bZIP)/ThioMac-PU.1-ChIP-Seq (GSE21512)/Homer	22.44%	1.00E−21
BHLHA15R	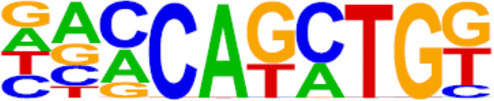		
	BHLHA15 (bHLH)/NIH3T3-BHLHB8.HA-ChIP-Seq (GSE119782)/Homer	22.44%	1.00E−04
NEUROG2	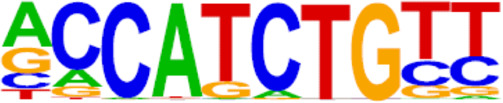		
	NeuroG2 (bHLH)/Fibroblast-NeuroG2-ChIP-Seq (GSE75910)/Homer	22.12%	1.00E−02
ATF3	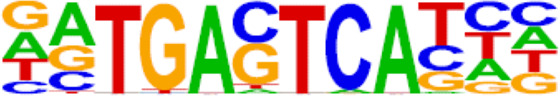		
	Atf3 (bZIP)/GBM-ATF3-ChIP-Seq (GSE33912)/Homer	21.15%	1.00E−22
SOX15	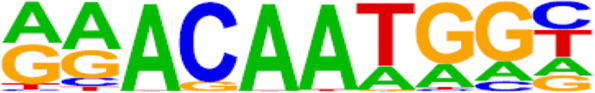		
	Sox15 (HMG)/CPA-Sox15-ChIP-Seq (GSE62909)/Homer	20.83%	1.00E−09
BATF	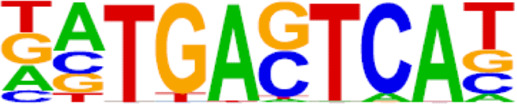		
	BATF (bZIP)/Th17-BATF-ChIP-Seq (GSE39756)/Homer	20.51%	1.00E−21

TFs, transcription factors; PIS, progestin-insensitive; PS, progestin-sensitive.

### Validation of candidate genes in the expanded samples

Endometrial samples from the Construction Group were analyzed by real-time quantitative PCR (RT-qPCR) for the expression of the twenty-five candidate genes. Each gene was analyzed in triplicate and normalized to the housekeeping gene *GAPDH*. Detailed primer sequences were listed in Supplementary Table S1. The value of the Δ cycle threshold (ΔCT) was used as the relative expression level of mRNA of the candidate genes compared to *GAPDH*. Then ΔCT values were normalized by SPSS Version 22.0 for subsequent analysis.

### Statistics

Statistical analysis was calculated using GraphPad Prism Version 8.0 and SPSS Version 22.0. RT-qPCR data were presented as the mean ± standard error of the mean (SEM) and were calculated by unpaired *t* test, unless otherwise noted. A two-tailed *p* value less than 0.05 was considered statistically significant.

To determine which candidate genes could be used for predicting progestin insensitivity, predictive models were established using multinomial logistic regression (SPSS Version 22.0). The PS-C, sub-PS-C, and PIS-C groups were identified as the dependent variables. Normalized ΔCT values of candidate genes were stratified into low, medium, and high expression stratifications according to cutoff values (X-tile Version 3.6). Then, the expression stratification of candidate genes was identified as an independent variable. The PS-C group was regarded as the control group in the multinomial logistic regression method. The predictive accuracy of the established models to predict PS, sub-PS and PIS was analyzed. Model fitting was used to illustrate the reliability of the models.

### Availability of supporting data

The raw data and processed data used in this study have been uploaded to the Gene Expression Omnibus repository under GEO accession number GSE201928 at https://www.ncbi.nlm.nih.gov/geo/.

## Results

### Comparison of chromatin accessibility between PIS and PS cases by ATAC-Seq

**FIGURE 1:**
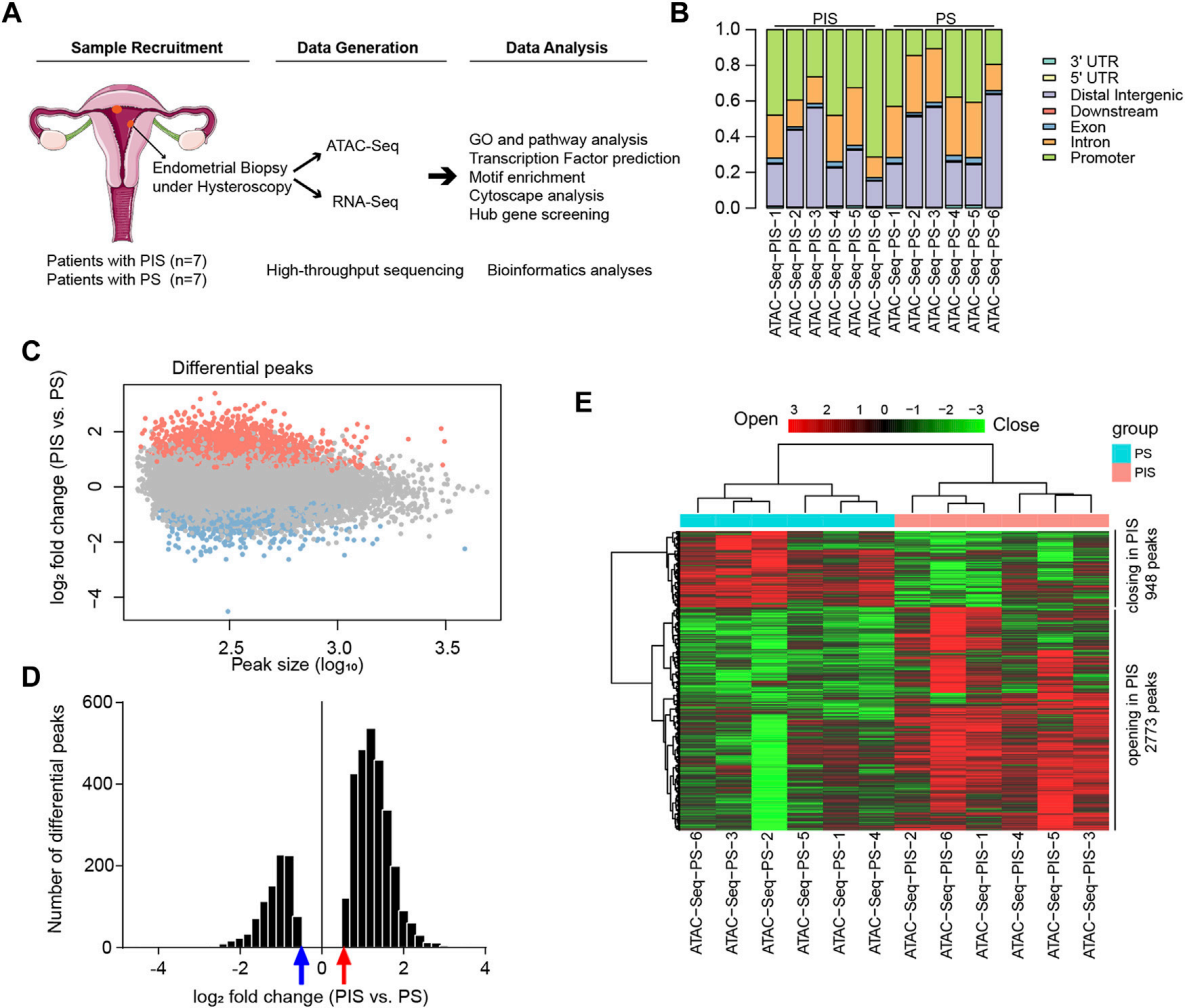
Landscape of genomic chromatin accessibility by ATAC-Seq. **(A)** Flowchart of study design. Endometrial lesions in the Analysis Group were collected for ATAC-Seq and RNA-Seq and further data analysis. **(B)** Genomic distribution of differential peaks. Bars with different colors and lengths represent different elements in the human genome and proportions, respectively. **(C)** Scatter plot of the chromatin accessibility at each peak in the PIS group compared to the PS group. The X-axis represents the peak size (log_10_), and the Y-axis represents the log_2_ fold change (PIS vs. PS) in ATAC-Seq analysis. The orange-red dots represent the opening peaks and the light blue dots represent the closing peaks in the PIS group compared to the PS group. **(D)** The histogram presents the distribution of log_2_ fold change of the differential peaks (PIS vs. PS). The abscissa represents log_2_ fold change of the differential peaks (PIS vs. PS) and the vertical axis represents the number of the differential peaks. Red arrow indicates log_2_ fold change = 0.5849 while blue arrow indicates log_2_ fold change = −0.5849. **(E)** Hierarchical cluster analysis of all the regulated opening and closing peaks in genes. Red plates represent opening peaks, while green plates indicate closing peaks in the PIS and PS groups. Abbreviations: PIS, progestin insensitive; PS, progestin sensitive; ATAC-Seq, assay for transposase-accessible chromatin sequencing; RNA-Seq, RNA sequencing; UTR, untranslated region.

Flowchart of study design was shown in Figure 1A. Firstly, genomic chromatin accessibility was analyzed by ATAC-Seq using samples from the Analysis Group (PIS, *n* = 7 and PS, *n* = 7). Five patients from each group had both ATAC-Seq and RNA-Seq data. The remaining two patients in each group had only ATAC-Seq data or RNA-Seq data, respectively. In the ATAC-Seq results, the proportion of all reads in each sample was matched to the elements in the human genome according to functional and positional information. The accessibility of transcriptional sites was more abundant in the promoter region in the PIS group but more abundant in intron and distal intergenic sites in the PS group (Figure 1B). The accessibility of the other four sites, including the 3′ UTR, 5’ UTR, downstream and exon, constituted a very small percentage of accessible transcriptional sites. After ATAC-Seq analysis, approximately 3721 differential opening or closing peaks were enriched, and most peaks were between 10^^2^ and 10^^3^ in size (Figure 1C). Additionally, distribution of 3721 differential peaks [log_2_ fold change (PIS vs. PS)] were provided, and the results showed that PIS group had more opening differential peaks than the PS group (Figure 1D). In detail, 2773 opening peaks and 948 closing peaks were shown in the PIS group compared to the PS group by hierarchical cluster analysis (Figure 1E). The heatmap showed that a higher proportion of genes were transcriptionally active in PIS cases than in PS cases. Gene peaks in six samples of each group were hierarchically clustered into one group, illustrating the reliability and accuracy of ATAC-Seq data.

### Comparison of expression profiles between PIS and PS cases by RNA-Seq

**FIGURE 2:**
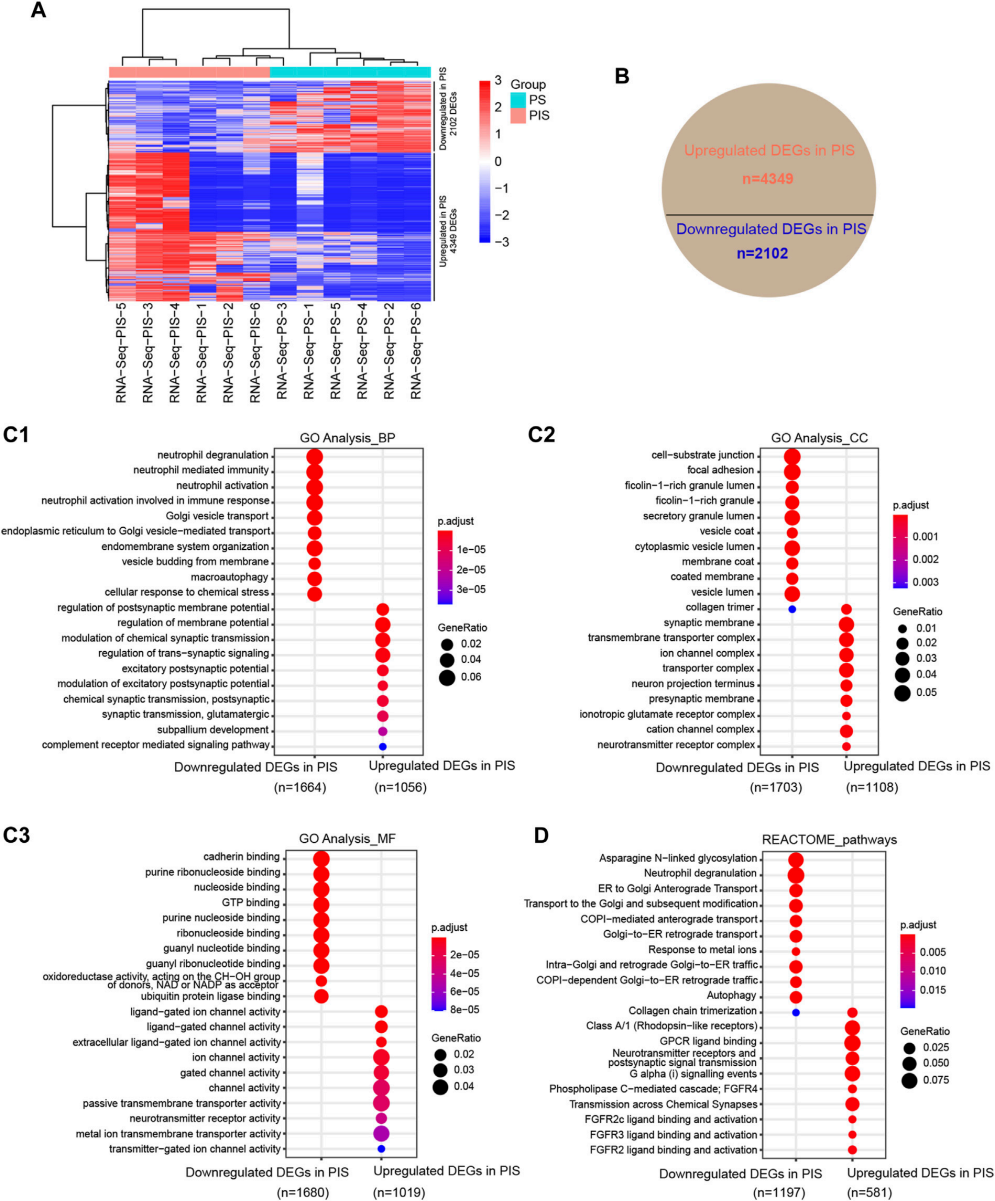
Expression profiles by RNA-Seq in PIS and PS patients with EAH and EEC. **(A)** Hierarchical cluster analysis of all DEGs annotated by FPKM by using DESeq2 normalization. The rows represent the 4349 upregulated and 2102 downregulated genes. Red grids represent upregulated genes while blue grids represent downregulated genes. **(B)** Statistical pie chart of upregulated and downregulated DEGs in the PIS group compared to the PS group. **(C)** Bubble diagram of the GO analysis of the upregulated and downregulated DEGs in the PIS group compared to the PS group, including BP (**C1**), CC (**C2**), and MF (**C3**). The top ten clusters with adjusted *p* < 0.05 were shown. **(D)** REACTOME pathway annotation of upregulated and downregulated DEGs in the PIS group compared to the PS group. The top ten enriched pathways with adjusted *p* < 0.05 were shown. Abbreviations: PIS, progestin insensitive; PS, progestin sensitive; DEGs, differentially expressed genes; GO, Gene Ontology; BP, biological process; CC, cellular components; MF, molecular function.

To compare the expression profiles between PIS and PS lesions in the Analysis Group, RNA-Seq was conducted and analyzed. DEGs were shown by hierarchical cluster analysis (Figure 2A). There were 4349 upregulated and 2102 downregulated DEGs in the PIS group compared to the PS group (Figure 2B). To identify whether these upregulated and downregulated DEGs in the PIS group were enriched in particular functions, GO annotation, including BP, CC, and MF categories, was performed (Figures 2C1,C2,C3). In the BP categories, downregulated DEGs in the PIS group were mainly enriched in neutrophil-associated activity, Golgi vesicle transport, endomembrane system organization, macroautophagy, and cellular response to chemical stress, while upregulated DEGs in the PIS group were mainly enriched in membrane potential and synaptic signaling-related functions (Figure 2C1). In accordance with the results for the BP categories, the CC categories showed that downregulated DEGs in the PIS group were mainly enriched in granule lumen, vesicle lumen, cell-substrate junction, and focal adhesion, while upregulated DEGs in the PIS group were enriched in synaptic membrane and related transporter complex (Figure 2C2). In the MF categories, downregulated DEGs in the PIS group were enriched in various kinds of binding, including cadherin, nucleoside, GTP, and ubiquitin protein ligase binding, etc., while upregulated DEGs in the PIS group were enriched in channel, transmembrane transporter, and neurotransmitter receptor activity (Figure 2C3). Furthermore, REACTOME pathway annotation of the DEGs showed that downregulated DEGs in the PIS group were significantly enriched in pathways including asparagine N-linked glycosylation, neutrophil degranulation, autophagy, and transport between Golgi and endoplasmic reticulum (ER), while the upregulated DEGs in the PIS group were enriched in chemical and synaptic signal transmission, fibroblast growth factor receptor (FGFR), and G protein-coupled receptor (GPCR) (Figure 2D). RNA-Seq data demonstrated that expression profiles varied widely between PIS and PS cases.

### Gene ontology and REACTOME analysis by ATAC-Seq and RNA-Seq integration

**FIGURE 3:**
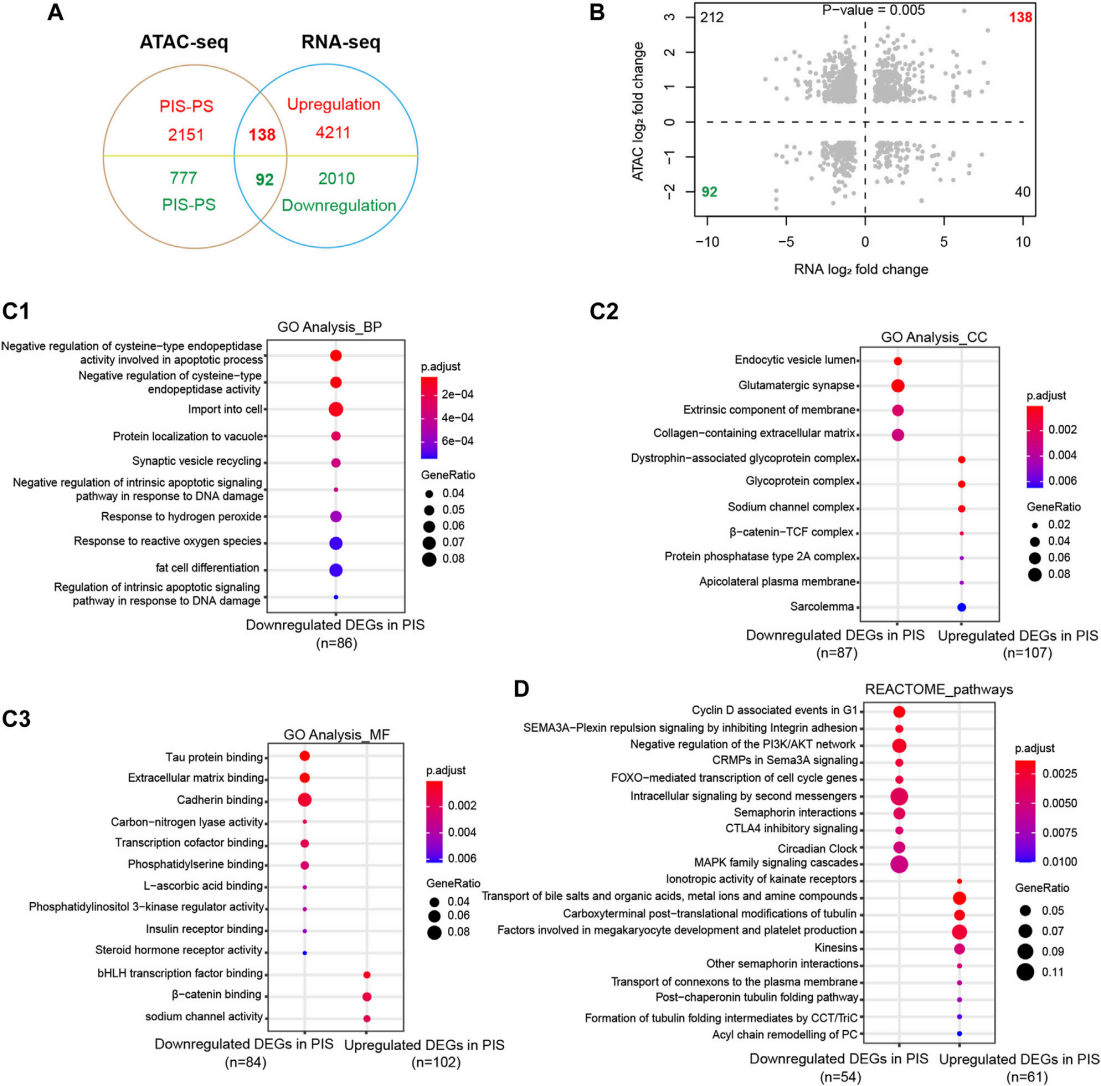
Enrichment analysis of DEGs integrated by ATAC-Seq and RNA-Seq. **(A)** Venn diagram of DEGs in RNA-Seq with differential opening and closing peaks in ATAC-Seq. **(B)** Chromatin accessibility correlates significantly with the 230 overlapping DEGs (Pearson analysis, *p* = 0.005). Dashed lines delineate the set of DEGs in RNA-Seq (X-axis) and differential opening or closing peaks in ATAC-Seq (Y-axis) between the PIS and PS groups. Shaded points in the upper right quadrant and lower left quadrant define the genes showing congruent chromatin accessibility and gene expression. **(C)** GO annotation of the upregulated and downregulated DEGs in the PIS group compared to the PS group was performed based on ATAC-Seq and RNA-Seq integration, including BP (**C1**), CC (**C2**), and MF (**C3**). The top ten clusters with adjusted *p* < 0.05 were shown. **(D)** REACTOME pathway annotation of the overlapping upregulated and downregulated DEGs in the PIS group by ATAC-Seq and RNA-Seq integration. The top ten enriched pathways with adjusted *p* < 0.05 were shown. Abbreviations: ATAC-Seq, assay for transposase-accessible chromatin sequencing; RNA-Seq, RNA sequencing; PIS, progestin insensitive; PS, progestin sensitive; DEGs, differentially expressed genes; GO, Gene Ontology; BP, biological process; CC, cellular components; MF, molecular function.

To further determine the specific functions and pathways related to progestin insensitivity, ATAC-Seq and RNA-Seq results were integrated for further analysis. By overlapping the results of ATAC-Seq and RNA-Seq, the PIS group had 138 upregulated DEGs with opening peaks and 92 downregulated DEGs with closing peaks in chromatin accessibility compared to the PS group (Figure 3A). Correlation analysis showed a significant positive correlation between expression profiles and chromatin accessibility of the above mentioned 230 overlapping DEGs (Figure 3B). To gain further insight into whether these 230 overlapping DEGs were engaged in specific functions and pathways, GO annotation and REACTOME pathways were performed. In BP categories, the overlapping downregulated DEGs in the PIS group mainly influenced cell import-transportation, negative regulation of cysteine-type endopeptidase activity, response to reactive oxygen species and fat cell differentiation (Figure 3C1). In CC categories, overlapping downregulated DEGs in the PIS group were enriched in glutamatergic synapse, extrinsic component of membrane, collagen-containing extracellular matrix, and endocytic vesicle lumen, while those overlapping upregulated DEGs in the PIS group were located in glycoprotein complex, sodium channel complex, β-catenin-TCF complex, and sarcolemma (Figure 3C2). Similarly, in MF categories, overlapping downregulated DEGs in the PIS group were enriched in extracellular matrix binding, cadherin binding, transcriptional cofactor binding and phosphatidylserine binding, while upregulated DEGs in the PIS group were associated with bHLH transcription factor binding, β-catenin binding, and sodium channel activity (Figure 3C3). Furthermore, REACTOME pathway analysis showed that these overlapping downregulated DEGs in the PIS group mainly influenced pathways including MAPK family signaling cascades, intracellular signaling by second messengers, negative regulation of the PI3K/AKT network, cyclin D-associated events in G1, and FOXO−mediated transcription of cell cycle genes, while upregulated DEGs in the PIS group were enriched in pathways including transport of bile salts and organic acids, metal ions and amine compounds, carboxyterminal post-translational modifications of tubulin, factors involved in megakaryocyte development and platelet production, and kinesins (Figure 3D). Taken together, these data suggested that the overlapping downregulated DEGs in the PIS group are responsible for signal transfer, the activity of transcription cofactors, DNA damage, cell apoptosis and cell cycle, while the overlapping upregulated DEGs in the PIS group are mainly responsible for substance transport and the regulation of cytoskeletal proteins.

### Screening of candidate genes for predicting progestin insensitivity

**FIGURE 4:**
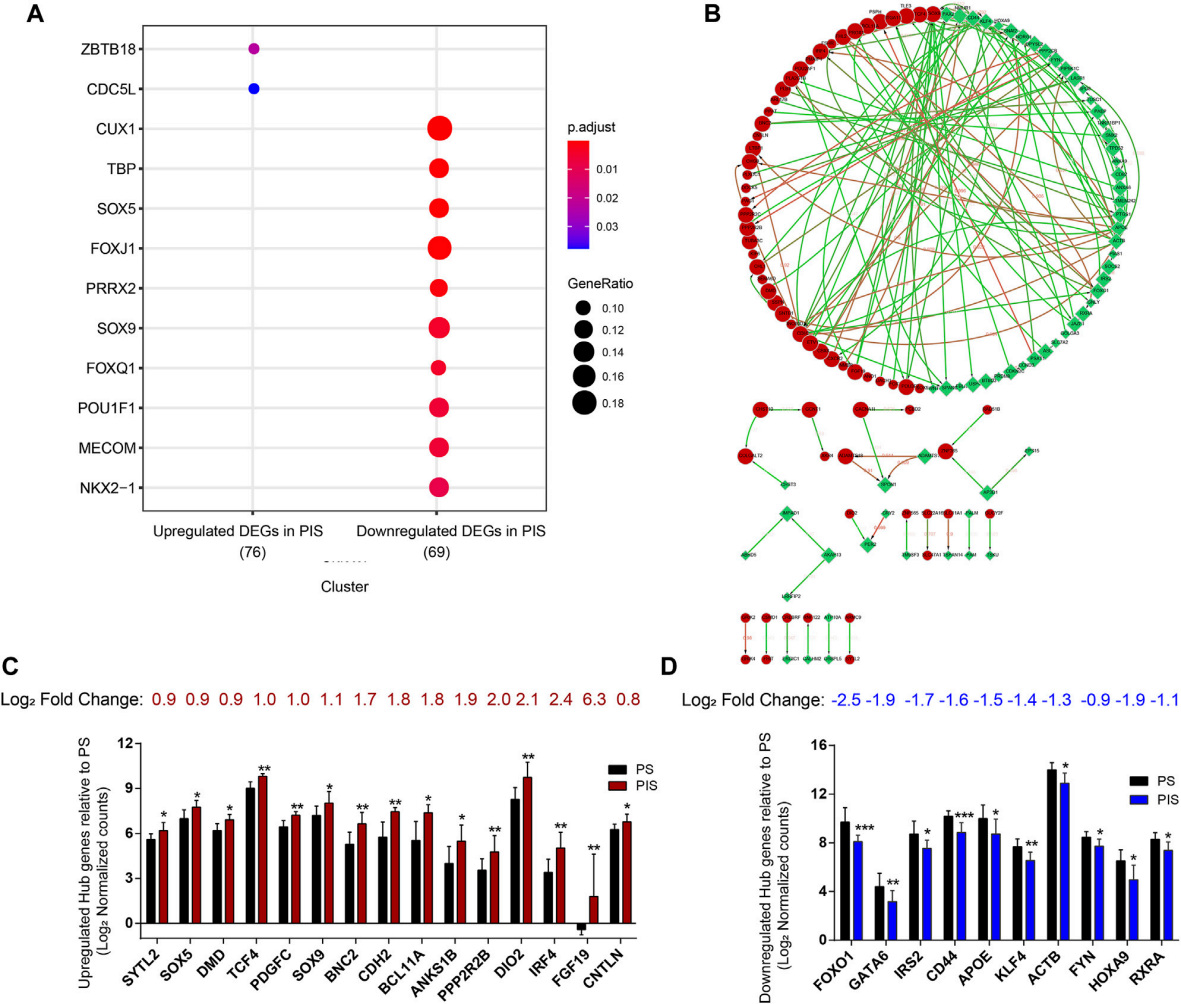
Bioinformatics analyses for screening the potential candidate genes. **(A)** Bubble diagram showing the transcription factor-binding sites clustered based on 230 overlapping DEGs. **(B)** Protein interaction networks were analyzed through STRING and Cytoscape analysis. Red nodes represent the upregulated overlapping DEGs, and green nodes represent the downregulated overlapping DEGs in the PIS group compared to the PS group. The larger the node is, the higher the connectivity is. Edges between two nodes indicate potential interactions between two proteins encoded by corresponding DEGs. The higher the value around the edges between connected nodes is, the higher the credibility is. **(C)** Log_2_ normalized counts of 15 upregulated candidate genes in the PIS group relative to the PS group. **(D)** Log_2_ normalized counts of 10 downregulated candidate genes in the PIS group relative to the PS group. Abbreviations: DEGs, differentially expressed genes; PIS, progestin insensitive; PS, progestin sensitive.

To further screen candidate genes predicting progestin insensitivity, potential TFs that regulate the expression of the 230 overlapping DEGs were enriched by HOMER Software. The TFs identified included CUX1, TBP, SOX5, FOXJ1, PRRX2, SOX9, FOXQ1, POU1F1, MECOM, and NKX2-1 based on the 69 downregulated DEGs in the PIS group, while only ZBTB18 and CDC5Lwereidentified based on 76 upregulated DEGs in the PIS group (Figure 4A). Additionally, motif enrichment was performed by homer peak analysis based on the results of ATAC-Seq and RNA-Seq integration. The generated homer known TFs with more than 20% of target sequences with motifs enriched in chromatin regions (PIS vs. PS) included NANOG, TGIF2, NF1, HOXA9, FOXO1, SP2, SOX10, SOX3, TWIST2, SOX6, SOX21, KLF5, MAZ, TCF4, AP-1, BHLHA15R, NEUROG2, ATF3, SOX15, and BATF (Table 2). Additionally, the interactions between proteins encoded by DEGs were analyzed using STRING and Cytoscape software (Figure 4B). Potential candidate genes or central genes were screened out based on the principle that more connected lines had higher combined scores. The left part showed the proteins encoded by the upregulated DEGs in the PIS group, and the top four social proteins with more than 4 connected lines were encoded by *SOX9*, *CDH2*, *IRF4*, and *TCF4*, respectively. There were eight proteins in the right part that had more than 4 connected lines in the downregulated DEGs in the PIS group, which were encoded by *CD44*, *ACTB*, *KLF4*, *APOE*, *SNAI2*, *FYN*, *PAX2*, and *FOXO1*, respectively. Finally, twenty-five candidate genes (*SYTL2*, *SOX5*, *DMD*, *TCF4*, *PDGFC*, *SOX9*, *BNC2*, *CDH2*, *BCL11A*, *ANKS1B*, *PPP2R2B*, *DIO2*, *IRF4*, *FGF19*, *FOXO1*, *GATA6*, *IRS2*, *CD44*, *APOE, KLF4*, *ACTB*, *FYN*, *CNTLN*, *HOXA9*, and *RXRA*.) were screened out based on bioinformatics analyses and literature review. The expression levels of these genes were presented according to the RNA-Seq results (Figures 4C,D).

### Establishment of potential models for predicting progestin insensitivity

Samples from the Construction Group (*n* = 35) were used for model construction. To construct models that can precisely predict the status of progestin sensitivity, 35 cases were further classified as PS-C (*n* = 13), sub-PS-C (*n* = 15) and PIS-C (*n* = 7), as shown in Table 1. Firstly, RT-qPCR was used to determine the expression of the 25 candidate genes in these 35 cases. As the CT values of *SYTL2*, *ANKS1B*, *PPP2R2B*, and *FGF19* exceeded 35, which suggested low gene expression and inaccurate analyses, these four genes were not included in the following analyses.

Predictive models were established by using multinomial logistic regression based on normalized ΔCT values of the remaining 21 genes according to different progestin sensitive conditions. The results in Table 3 showed that a total of 25 predictive models were generated with predictive accuracy of 100% for PIS-C patients, among which 11 models had predictive accuracy of more than 80% for sub-PS-C prediction (*p* < 0.01). Three models’ overall predictive accuracy were higher than 90%, involving 9 candidate genes (*FOXO1*, *IRS2*, *PDGFC*, *DIO2*, *SOX9*, *BCL11A*, *APOE*, *FYN*, and *KLF4*) (Supplementary Figure S1).

**TABLE 3:** Characteristics of different predictive models based on candidate genes.

No.	Candidate genes included in models	Model fitting (*p* value)	Pseudo R square	Predictive accuracy of PIS-C (%)	Predictive accuracy of sub-PS-C (%)	Predictive accuracy of sub-PS-C (%)	Overall accuracy of prediction (%)
1	BCL11A + SOX9+ApoE + FOXO1+FYN + KLF4+DIO2	<0.001	≥0.846	100	93.3	84.6	91.4
2	BCL11A + SOX9+ApoE + FOXO1+FYN + KLF4+IRS2+DIO2	<0.001	≥0.857	100	86.7	100.0	94.3
3	BCL11A + PDGFC + SOX9+ApoE + FYN + KLF4+IRS2+DIO2	<0.001	≥0.846	100	86.7	92.3	91.4
4	BCL11A + PDGFC + ApoE + FOXO1+FYN + KLF4+DIO2	<0.01	≥0.793	100	80.0	92.3	88.6
5	BCL11A + PDGFC + ApoE + FOXO1+FYN + KLF4+IRS2+DIO2	<0.01	≥0.828	100	80.0	92.3	88.6
6	BCL11A + ApoE + FOXO1+FYN + KLF4+DIO2	<0.001	≥0.743	100	80.0	84.6	85.7
7	BCL11A + SOX9+ApoE + FYN + KLF4+DIO2	<0.001	≥0.798	100	80.0	84.6	85.7
8	BCL11A + PDGFC + ApoE + FOXO1+FYN + IRS2+DIO2	<0.001	≥0.811	100	80.0	84.6	85.7
9	BCL11A + ApoE + FOXO1+FYN + KLF4+IRS2+DIO2	<0.01	≥0.748	100	80.0	76.9	82.9
10	BCL11A + PDGFC + SOX9+ApoE + FOXO1+KLF4+DIO2	<0.01	≥0.812	100	80.0	76.9	82.9
11	BCL11A + ApoE + FYN + KLF4+IRS2+DIO2	<0.01	≥0.698	100	80.0	69.2	80.0
12	BCL11A + PDGFC + ApoE + FYN + KLF4+IRS2+DIO2	<0.01	≥0.787	100	73.3	92.3	85.7
13	BCL11A + PDGFC + ApoE + FOXO1+FYN + DIO2	<0.01	≥0.761	100	73.3	92.3	85.7
14	BCL11A + PDGFC + ApoE + FOXO1+KLF4+DIO2	<0.01	≥0.602	100	73.3	84.6	82.9
15	BCL11A + ApoE + FYN + KLF4+DIO2	<0.01	≥0.682	100	73.3	76.9	80.0
16	BCL11A + SOX9+ApoE + FYN + KLF4+IRS2+DIO2	<0.01	≥0.812	100	73.3	100	88.6
17	BCL11A + PDGFC + FOXO1+KLF4+IRS2+DIO2	<0.01	≥0.61	100	66.7	84.6	80.0
18	BCL11A + ApoE + FOXO1+FYN + IRS2+DIO2	<0.01	≥0.675	100	66.7	76.9	77.1
19	BCL11A + PDGFC + FYN + KLF4+IRS2+DIO2	<0.001	≥0.726	100	60.0	92.3	80.0
20	BCL11A + ApoE + FYN + IRS2+DIO2	<0.01	≥0.634	100	60.0	86.4	77.1
21	PDGFC + ApoE + FOXO1+FYN + KLF4+DIO2	<0.05	≥0.528	100	53.3	69.2	68.6
22	ApoE + FOXO1+FYN + KLF4+IRS2+DIO2	<0.05	≥0.526	100	53.3	69.2	68.6
23	ApoE + FYN + KLF4+IRS2+DIO2	<0.05	≥0.479	100	46.7	61.5	62.9
24	PDGFC + FYN + KLF4+IRS2+DIO2	<0.05	≥0.457	100	40.0	76.9	65.7
25	PDGFC + ApoE + FYN + KLF4+DIO2	<0.05	≥0.480	100	40.0	69.2	62.9

No., number; PIS-C, progestin-insensitive in Construction Group; sub-PS-C, progestin-sub-sensitive in Construction Group; PS-C, progestin-sensitive in Construction Group.

## Discussion

It is necessary to establish highly accurate predictive models for identifying PIS patients and helping provide individualized fertility-preserving treatment for EAH and EEC patients. In this study, through ATAC-Seq and RNA-Seq analyses of 14 cases and verification of candidate genes in 35 expanded samples, predictive models comprising nine genes (*FOXO1*, *IRS2*, *PDGFC*, *DIO2*, *SOX9*, *BCL11A*, *APOE*, *FYN*, and *KLF4*) were established. Our models provided new molecular markers that could be used in combination with the well-known PR status to help identify PIS patients prior to treatment initiation.

In this study, we found that the expression of *PDGFC*, *DIO2*, *SOX9*, and *BCL11A*was upregulated and *FOXO1*, *IRS2*, *APOE, FYN* and *KLF4* was downregulated in PIS endometrial lesions compared with PS endometrial lesions. These nine genes were all reported to play important roles in tumor progression or drug response. *PDGFC-*encoded platelet-derived growth factor C was reported to promote angiogenesis, cancer cell proliferation, invasion, and metastasis (Kim et al., 2021). *SOX9-* and *BCL11A*-encoded proteins were both involved in inducing tumor initiation, proliferation, migration, and chemoresistance (Yin et al., 2019; Jana et al., 2020). *DIO2*-encoded protein can catalyze the conversion of tetraiodothyronine to bioactive triiodothyronine. Triiodothyronine was reported to be associated with lipid accumulation and metabolism in adipose tissue, which contributes to obesity-related insulin resistance (Bradley et al., 2018). Previous studies showed that high expression levels of PDGFC, SOX9, BCL11A, and DIO2 were associated with poor response to chemotherapy in cancer cells and short survival time of various patients, which could be regarded as negative prognostic factors (Bradley et al., 2018; Yin et al., 2019; Jana et al., 2020; Kim et al., 2021). Carriers of the *DIO2* polymorphism were also reported to be predisposed to the development of endometrial cancer (Janowska et al., 2022). Furthermore, the inhibition of *SOX9* or *DIO2* has been reported to be a potential therapeutic strategy for cancer (Carrasco-Garcia et al., 2019; Kojima et al., 2019).

*FOXO1*, an important member of the *FOXO* subfamily in the *FOX* family, encodes a transcription factor and has been reported to be involved in various physiological processes, including inducing cancer cell cycle arrest and suppressing the migration and invasion of cancer cells (Xing et al., 2018). *FOXO1* was also identified as a progesterone target gene containing PR elements within the promoter regions (Yang et al., 2011). Downregulated FOXO1expression was found in progestin-resistant EC cells and was associated with progestin insensitivity in EC patients (Yang et al., 2011; Reyes et al., 2016; Wang et al., 2018b). *IRS2*, encoding a kind of insulin receptor substrate that is commonly phosphorylated by the receptor tyrosine kinase, was reported to promote cell proliferation, invasion and sphere formation of cancer cells (Shaw, 2011). However, *IRS2* amplification and high expression of IRS2 were potentially related to good response to chemotherapy (Lee et al., 2020). *APOE*, one of apolipoproteins, plays anti-immunosuppressive and anti-metastatic roles in tumorigenesis (Tavazoie et al., 2018). High expression of APOE was reported to be associated with good prognosis of thyroid cancer patients (Nan et al., 2021). *KLF4* encodes a transcription factor that acted as a tumor suppressor which inhibited cell cycle, promoted apoptosis and differentiation, and suppressed metastasis (Yan et al., 2016). Downregulated expression of KLF4 by promoter methylation modification was reported in EC tissues, which was associated with accelerated tumorigenesis, drug resistance and poor prognosis (Jia et al., 2012; Danková et al., 2018). *FYN* encodes a membrane-associated tyrosine kinase that promoted cell proliferation, migration and invasion and inhibited apoptosis of cancer cells (Saito et al., 2010). Overexpression of FYN was reported to be correlated with chemotherapy resistance and poor survival (Elias et al., 2014). However, the roles of 9 candidate genes in regulating progestin response needs further investigation.

The strength of our study is the use of ATAC-Seq together with RNA-Seq technology to help identify the upregulated or downregulated genes with simultaneous opening or closing chromatin accessibility which effectively improves the accuracy of candidate gene screening. The improved ATAC-Seq protocol used in this work could further reduce background disturbances from different individuals to improve the accuracy of the analysis (Corces et al., 2017). Thirty-five patients with various progestin sensitive conditions were used for further data verification and construction of potential predictive models with an overall predictive accuracy above 90%. There are some limitations in the study. First, the sample size was not large enough to address tissue heterogeneity. Second, integration of ATAC-Seq and RNA-Seq can be used to analyze the epigenetic and transcriptional changes in genes, but post-transcriptional and post-translational regulatory levels cannot be analyzed.

In conclusion, the predictive models we provided may be useful in identifying progestin insensitive EAH and EEC patients before initiating fertility-sparing therapy. The accuracy of our predictive models requires more samples validation and molecular mechanism exploration.

## Data Availability

The data presented in the study are deposited in the Gene Expression Omnibus repository, accession number GSE201928 at https://www.ncbi.nlm.nih and we have released the accession.

## References

[B1] Bradley D. Liu J. Blaszczak A. Wright V. Jalilvand A. Needleman B. (2018). Adipocyte DIO2 expression increases in human obesity but is not related to systemic insulin sensitivity. J. Diabetes Res. 2018, 2464652. 10.1155/2018/2464652 30116736 PMC6079440

[B2] Buenrostro J. D. Wu B. Chang H. Y. Greenleaf W. J. (2015). ATAC-seq: A method for assaying chromatin accessibility genome-wide. Curr. Protoc. Mol. Biol. 109 (21), 21–29. 10.1002/0471142727.mb2129s109 PMC4374986 25559105

[B3] Carrasco-Garcia E. Álvarez-Satta M. García-Puga M. Ribeiro M. L. Arevalo S. Arauzo-Bravo M. (2019). Therapeutic relevance of SOX9 stem cell factor in gastric cancer. Expert Opin. Ther. Targets 23, 143–152. 10.1080/14728222.2019.1559826 30572738

[B4] Corces M. R. Trevino A. E. Hamilton E. G. Greenside P. G. Sinnott-Armstrong N. A. Vesuna S. (2017). An improved ATAC-seq protocol reduces background and enables interrogation of frozen tissues. Nat. Methods 14, 959–962. 10.1038/nmeth.4396 28846090 PMC5623106

[B5] Danková Z. BRANý D. DVORSKá D. ŇACHAJOVá M. Fiolka R. GRENDáR M. (2018). Methylation status of KLF4 and HS3ST2 genes as predictors of endometrial cancer and hyperplastic endometrial lesions. Int. J. Mol. Med. 42, 3318–3328. 10.3892/ijmm.2018.3872 30221668 PMC6202087

[B6] Elias D. Vever H. Lænkholm A. V. Gjerstorff M. F. Yde C. W. Lykkesfeldt A. E. (2014). Gene expression profiling identifies FYN as an important molecule in tamoxifen resistance and a predictor of early recurrence in patients treated with endocrine therapy. Oncogene 34, 1919–1927. 10.1038/onc.2014.138 24882577

[B7] Fan R. Wang W. Wei W. Zheng W. (2017). Mechanism of progestin resistance in endometrial precancer/cancer through Nrf2-survivin pathway. Am. J. Transl. Res. 9, 1483–1491. 28386373 PMC5376038

[B8] Gallos I. D. Yap J. Rajkhowa M. Luesley D. M. Coomarasamy A. Gupta J. K. (2012). Regression, relapse, and live birth rates with fertility-sparing therapy for endometrial cancer and atypical complex endometrial hyperplasia: A systematic review and metaanalysis. Am. J. Obstet. Gynecol. 207, 266–312. 10.1016/j.ajog.2012.08.011 23021687

[B9] Gunderson C. C. Fader A. N. Carson K. A. Bristow R. E. (2012). Oncologic and reproductive outcomes with progestin therapy in women with endometrial hyperplasia and grade 1 adenocarcinoma: A systematic review. Gynecol. Oncol. 125, 477–482. 10.1016/j.ygyno.2012.01.003 22245711

[B10] Jana S. Madhu Krishna B. Singhal J. Horne D. Awasthi S. Salgia R. (2020). SOX9: The master regulator of cell fate in breast cancer. Biochem. Pharmacol. 174, 113789. 10.1016/j.bcp.2019.113789 31911091 PMC9048250

[B11] Janowska M. Potocka N. Paszek S. Skrzypa M. Zulewicz K. Kluz M. (2022). An assessment of GPX1 (rs1050450), DIO2 (rs225014) and SEPP1 (rs7579) gene polymorphisms in women with endometrial cancer. Genes (Basel) 13, 188. 10.3390/genes13020188 35205233 PMC8871918

[B12] Jia Y. Zhang W. Liu H. Peng L. Yang Z. Lou J. (2012). Inhibition of glutathione synthesis reverses Krüppel-like factor 4-mediated cisplatin resistance. Cancer Chemother. Pharmacol. 69, 377–385. 10.1007/s00280-011-1708-7 21833590

[B13] Kim S. You D. Jeong Y. Yoon S. Y. Kim S. A. Lee J. E. (2021). Inhibition of platelet-derived growth factor C and their receptors additionally increases doxorubicin effects in triple-negative breast cancer cells. Eur. J. Pharmacol. 895, 173868. 10.1016/j.ejphar.2021.173868 33460613

[B14] Kojima Y. Kondo Y. Fujishita T. Mishiro‐Sato E. Kajino‐Sakamoto R. Taketo M. M. (2019). Stromal iodothyronine deiodinase 2 ( DIO 2) promotes the growth of intestinal tumors in Apc Δ716 mutant mice. Cancer Sci. 110, 2520–2528. 10.1111/cas.14100 31215118 PMC6676103

[B15] Lee M. S. Jung K. Song J. Y. Sung M. J. Ahn S. B. Lee B. (2020). IRS2 amplification as a predictive biomarker in response to ceritinib in small cell lung cancer. Mol. Ther. - Oncolytics 16, 188–196. 10.1016/j.omto.2019.12.009 32099898 PMC7029374

[B16] Love M. I. Huber W. Anders S. (2014). Moderated estimation of fold change and dispersion for RNA-seq data with DESeq2. Genome Biol. 15, 550. 10.1186/s13059-014-0550-8 25516281 PMC4302049

[B17] Nan B.-Y. Xiong G.-F. Zhao Z.-R. Gu X. Huang X.-S. Fedele M. (2021). Comprehensive identification of potential crucial genes and miRNA-mRNA regulatory networks in papillary thyroid cancer. BioMed Res. Int. 2021, 1–25. 10.1155/2021/6752141 33521130 PMC7817291

[B18] Raffone A. Travaglino A. Saccone G. Mollo A. De Placido G. Insabato L. (2019). Should progesterone and estrogen receptors be assessed for predicting the response to conservative treatment of endometrial hyperplasia and cancer? A systematic review and meta‐analysis. Acta Obstet. Gynecol. Scand. 98, 976–987. 10.1111/aogs.13586 30779338

[B19] Reyes H. D. Carlson M. J. Devor E. J. Zhang Y. Thiel K. W. Samuelson M. I. (2016). Downregulation of FOXO1 mRNA levels predicts treatment failure in patients with endometrial pathology conservatively managed with progestin-containing intrauterine devices. Gynecol. Oncol. 140, 152–160. 10.1016/j.ygyno.2015.10.023 26524723 PMC4784706

[B20] Saito Y. D. Jensen A. R. Salgia R. Posadas E. M. (2010). Fyn. Cancer 116, 1629–1637. 10.1002/cncr.24879 20151426 PMC2847065

[B21] Shaw L. M. (2011). The insulin receptor substrate (IRS) proteins. Cell Cycle 10, 1750–1756. 10.4161/cc.10.11.15824 21597332 PMC3142458

[B22] Siegel R. L. Miller K. D. Fuchs H. E. Jemal A. (2022). Cancer statistics, 2022. CA A Cancer J. Clin. 72, 7–33. 10.3322/caac.21708 35020204

[B23] Tavazoie M. F. Pollack I. Tanqueco R. Ostendorf B. N. Reis B. S. Gonsalves F. C. (2018). LXR/ApoE activation restricts innate immune suppression in cancer. Cell 172, 825–840. e18. 10.1016/j.cell.2017.12.026 29336888 PMC5846344

[B24] Trapnell C. Williams B. A. Pertea G. Mortazavi A. Kwan G. van Baren M. J. (2010). Transcript assembly and quantification by RNA-Seq reveals unannotated transcripts and isoform switching during cell differentiation. Nat. Biotechnol. 28, 511–515. 10.1038/nbt.1621 20436464 PMC3146043

[B25] Trojano G. Olivieri C. Tinelli R. Damiani G. R. Pellegrino A. Cicinelli E. (2019). Conservative treatment in early stage endometrial cancer: A review. Acta Biomed. 90, 405–410. 10.23750/abm.v90i4.7800 31910163 PMC7233769

[B26] Wang L. Feng Z. Wang X. Wang X. Zhang X. (2010). DEGseq: an R package for identifying differentially expressed genes from RNA-seq data. Bioinformatics 26, 136–138. 10.1093/bioinformatics/btp612 19855105

[B27] Wang Y. Zeng X. Liu W. (2018a). De novo transcriptomic analysis during Lentinula edodes fruiting body growth. Gene 641, 326–334. 10.1016/j.gene.2017.10.061 29066302

[B28] Wang Y. Zhang L. Che X. Li W. Liu Z. Jiang J. (2018b). Roles of SIRT1/FoxO1/SREBP-1 in the development of progestin resistance in endometrial cancer. Arch. Gynecol. Obstet. 298, 961–969. 10.1007/s00404-018-4893-3 30206735

[B29] Wang Y. Zhou R. Zhang X. Liu H. Shen D. Wang J. (2021). Significance of serum and pathological biomarkers in fertility-sparing treatment for endometrial cancer or atypical hyperplasia: A retrospective cohort study. BMC Women's Health 21, 252. 10.1186/s12905-021-01383-5 34162378 PMC8223344

[B30] Westin S. N. Fellman B. Sun C. C. Broaddus R. R. Woodall M. L. Pal N. (2021). Prospective phase II trial of levonorgestrel intrauterine device: Nonsurgical approach for complex atypical hyperplasia and early-stage endometrial cancer. Am. J. Obstet. Gynecol. 224, 191–e15. 10.1016/j.ajog.2020.08.032 32805208 PMC7855308

[B31] Xing Y. Q. Li A. Yang Y. Li X. X. Zhang L. N. Guo H. C. (2018). The regulation of FOXO1 and its role in disease progression. Life Sci. 193, 124–131. 10.1016/j.lfs.2017.11.030 29158051

[B32] Yamazawa K. Hirai M. Fujito A. Nishi H. Terauchi F. Ishikura H. (2007). Fertility-preserving treatment with progestin, and pathological criteria to predict responses, in young women with endometrial cancer. Hum. Reprod. 22, 1953–1958. 10.1093/humrep/dem088 17449880

[B33] Yan Y. Li Z. Kong X. Jia Z. Zuo X. Gagea M. (2016). KLF4-Mediated suppression of CD44 signaling negatively impacts pancreatic cancer stemness and metastasis. Cancer Res. 76, 2419–2431. 10.1158/0008-5472.can-15-1691 26880805 PMC4876033

[B34] Yang B. Xu Y. Zhu Q. Xie L. Shan W. Ning C. (2019). Treatment efficiency of comprehensive hysteroscopic evaluation and lesion resection combined with progestin therapy in young women with endometrial atypical hyperplasia and endometrial cancer. Gynecol. Oncol. 153, 55–62. 10.1016/j.ygyno.2019.01.014 30674421

[B35] Yang B. Y. Gulinazi Y. Du Y. Ning C. C. Cheng Y. L. Shan W. W. (2020). Metformin plus megestrol acetate compared with megestrol acetate alone as fertility‐sparing treatment in patients with atypical endometrial hyperplasia and well‐differentiated endometrial cancer: A randomised controlled trial. BJOG Int. J. Obstet. Gy 127, 848–857. 10.1111/1471-0528.16108 31961463

[B36] Yang S. Thiel K. W. Leslie K. K. (2011). Progesterone: The ultimate endometrial tumor suppressor. Trends Endocrinol. Metabolism 22, 145–152. 10.1016/j.tem.2011.01.005 PMC4062362 21353793

[B37] Yin J. Xie X. Ye Y. Wang L. Che F. (2019). BCL11A: A potential diagnostic biomarker and therapeutic target in human diseases. Biosci. Rep. 39, BSR20190604. 10.1042/BSR20190604 31654056 PMC6851505

[B38] Zhang H. Yan L. Bai Y. Li C. Guo Q. Wang C. (2015). Dual-specificity phosphatase 6 predicts the sensitivity of progestin therapy for atypical endometrial hyperplasia. Gynecol. Oncol. 136, 549–553. 10.1016/j.ygyno.2014.11.008 25451692

[B39] Zhang Y. Parmigiani G. Johnson W. E. (2020). ComBat-seq: Batch effect adjustment for RNA-seq count data. Nar. Genom Bioinform 2, lqaa078. 10.1093/nargab/lqaa078 33015620 PMC7518324

[B40] Zhou S. Xu Z. Yang B. Guan J. Shan W. Shi Y. (2021). Characteristics of progestin-insensitive early stage endometrial cancer and atypical hyperplasia patients receiving second-line fertility-sparing treatment. J. Gynecol. Oncol. 32, e57. 10.3802/jgo.2021.32.e57 34085795 PMC8192233

