# Supplementary material for: Identification of potential models for predicting progestin insensitivity in patients with endometrial atypical hyperplasia and endometrioid endometrial cancer based on ATAC-Seq and RNA-Seq integrated analysis
Source: Front Genet. 2022 Aug 26;13:952083. doi: 10.3389/fgene.2022.952083 (PMC9459090; doi:10.3389/fgene.2022.952083)
Supplement: Supplementary file 1 [file Table1.DOCX]

**Supplementary Figure 1. Expression of 9 candidate genes engaged in potential predictive models**

The X-axis represents the different groups, including PS-C, sub-PS-C, and PIS-C, based on time to achieve CR. The Y-axis represents the relative expression level of the candidate genes. ΔCT refers to the CT value of the candidate genes minus the CT value of *GAPDH*. A larger ΔCT value indicates a lower gene expression level. Data are presented as the mean ± SEM. The numbers on each line represent the P value compared between the two groups. Abbreviations: PS-C, progestin sensitive in the Construction Group; sub-PS-C, progestin sub-sensitive in the Construction Group; PIS-C, progestin insensitive in the Construction Group.
