# Supplementary figures and images for: Identification of potential models for predicting progestin insensitivity in patients with endometrial atypical hyperplasia and endometrioid endometrial cancer based on ATAC-Seq and RNA-Seq integrated analysis
Source: Front Genet. 2022 Aug 26;13:952083. doi: 10.3389/fgene.2022.952083 (PMC9459090; doi:10.3389/fgene.2022.952083)

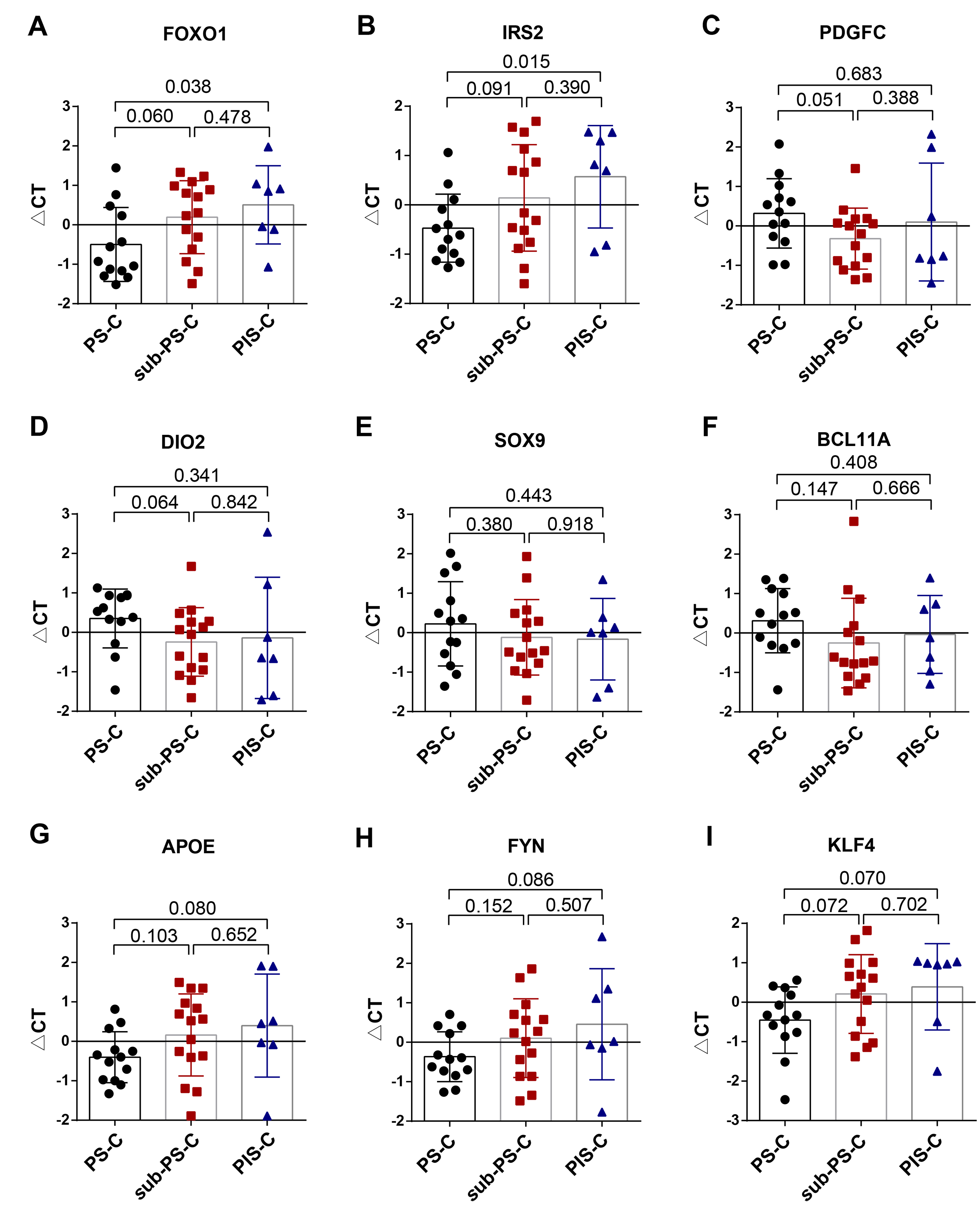

Supplement: Supplementary file 2 [file Image1.TIF]
