# Supplementary material for: Identification of potential models for predicting progestin insensitivity in patients with endometrial atypical hyperplasia and endometrioid endometrial cancer based on ATAC-Seq and RNA-Seq integrated analysis
Source: Front Genet. 2022 Aug 26;13:952083. doi: 10.3389/fgene.2022.952083 (PMC9459090; doi:10.3389/fgene.2022.952083)
Supplement: Supplementary file 3 [file Table2.DOCX]

**Supplementary Table 1. Primer sequences of the housekeeping and candidate genes.**

| **Gene Symbol** | **Primer Sequence (5' → 3')** | **Tm (℃)** |
| --- | --- | --- |
| GAPDH | F: GGGAAGGTGAAGGTCGGAGT | 59.5 |
|  | R: GGGGTCATTGATGGCAACA | 55.2 |
| SYTL2 | F: TGTGTTGAGCCTGAGCCATCTC | 59.5 |
|  | R: TGGAAGGCATTTTCCTAGCGGC | 59.5 |
| SOX5 | F: CTCGGCAAATGAAGGAGCAACTC | 59.6 |
|  | R: ACTGCCAGTTGCTGAGTCAGAC | 59.5 |
| DMD | F: GCTCAACCATCGATTTGCAGCC | 59.5 |
|  | R: TTCAGCCTCCAGTGGTTCAAGC | 59.5 |
| TCF4 | F: GCCTCTTCACAGTAGTGCCATG | 59.4 |
|  | R: GCTGGTTTGGAGGAAGGATAGC | 59.5 |
| PDGFC | F: TGAACCAGGGTTCTGCATCCAC | 59.5 |
|  | R: TAAGCAGGTCCAGTGGCAAAGC | 59.5 |
| SOX9 | F: AGGAAGCTCGCGGACCAGTAC | 61.5 |
|  | R: GGTGGTCCTTCTTGTGCTGCAC | 61.4 |
| BNC2 | F: ACTCTGCGGGACTATGTCCGAG | 61.4 |
|  | R: ACCGCAGAAACTGCTGAAGGGT | 59.5 |
| CDH2 | F: CCTCCAGAGTTTACTGCCATGAC | 59.6 |
|  | R: GTAGGATCTCCGCCACTGATTC | 59.5 |
| BCL11A | F: CTCGTTCTGCACATGGAGCTCT | 59.5 |
|  | R: GCAAGAGAAACCATGCACTGGTG | 59.6 |
| ANKS1B | F: ACAGGCAATCCAGCTCCTTCCA | 59.5 |
|  | R: CCAGTTCAATGGAATCCAGCCAC | 59.6 |
| PPP2R2B | F: ATGACTACCTCCGCAGCAAGCT | 59.5 |
|  | R: CATCACGCTTGGTGTTTCTGTCG | 59.6 |
| DIO2 | F: TTGAGCCGCTCCAAGTCCACTC | 61.4 |
|  | R: CTGTACTGGAGACATGCACCAC | 59.5 |
| IRF4 | F: GAACGAGGAGAAGAGCATCTTCC | 59.6 |
|  | R: CGATGCCTTCTCGGAACTTTCC | 59.5 |
| FGF19 | F: TGCACAGCGTGCGGTACCTCT | 61.5 |
|  | R: CGGTACACATTGTAGCCATCTGG | 59.6 |
| FOXO1 | F: CTACGAGTGGATGGTCAAGAGC | 59.5 |
|  | R: CCAGTTCCTTCATTCTGCACACG | 59.6 |
| GATA6 | F: GCCACTACCTGTGCAACGCCT | 61.5 |
|  | R: CAATCCAAGCCGCCGTGATGAA | 59.5 |
| IRS2 | F: CCTGCCCCCTGCCAACACCT | 63.6 |
|  | R: TGTGACATCCTGGTGATAAAGCC | 57.8 |
| CD44 | F: CCAGAAGGAACAGTGGTTTGGC | 59.5 |
|  | R: ACTGTCCTCTGGGCTTGGTGTT | 59.5 |
| APOE | F: GGGTCGCTTTTGGGATTACCTG | 59.5 |
|  | R: CAACTCCTTCATGGTCTCGTCC | 59.5 |
| KLF4 | F: CATCTCAAGGCACACCTGCGAA | 59.5 |
|  | R: TCGGTCGCATTTTTGGCACTGG | 59.5 |
| ACTB | F: CACCATTGGCAATGAGCGGTTC | 59.5 |
|  | R: AGGTCTTTGCGGATGTCCACGT | 59.5 |
| FYN | F: CTGGTCACCAAAGGAAGAGTGC | 59.5 |
|  | R: GGTCCTTTTTCCAGCAGTGGATC | 59.6 |
| CNTLN | F: GGTCGTCACCACACTGTTCTCA | 59.5 |
|  | R: CCAAAGTTTGAGAGGTCTGTGCT | 57.8 |
| HOXA9 | F: AGAATGAGAGCGGCGGAGACAA | 59.5 |
|  | R: CTCTTTCTCCAGTTCCAGGGTC | 59.5 |
| RXRA | F: TTGCCAAGCAGCCGACAAACAG | 59.5 |
|  | R: AAGGAGGCGATGAGCAGCTCAT | 59.5 |

Abbreviations: F, forward; R, reverse; Tm, melting temperature.
